# Supplementary material for: The Anti-Fibrotic Effects of CG-745, an HDAC Inhibitor, in Bleomycin and PHMG-Induced Mouse Models
Source: Molecules. 2019 Jul 31;24(15):2792. doi: 10.3390/molecules24152792 (PMC6696140; doi:10.3390/molecules24152792)
Supplement: Supplementary file 1 [file molecules-24-02792-s001.pdf]

Supplementary materials

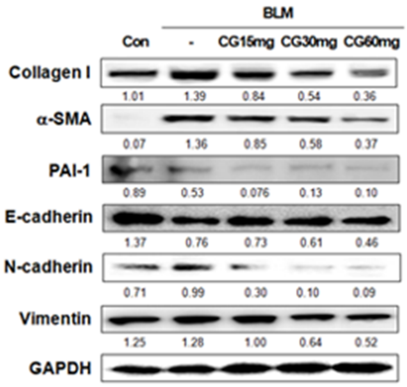

Supplementary Figure S1. CG-745 ameliorates pulmonary fibrosis induced by bleomycin in mice. Mice were instilled with bleomycin (2 mg/kg) intratracheally on day 0, and CG-745 (15, 30 or 60 mg/kg) was intraperitoneally administered daily for 14 days. The protein levels of fibrotic marker (collagen I, α-SMA, PAI-1) and EMT marker (E-cadherin, N-cadherin, vimentin) in the lung tissues were analyzed by western blotting.

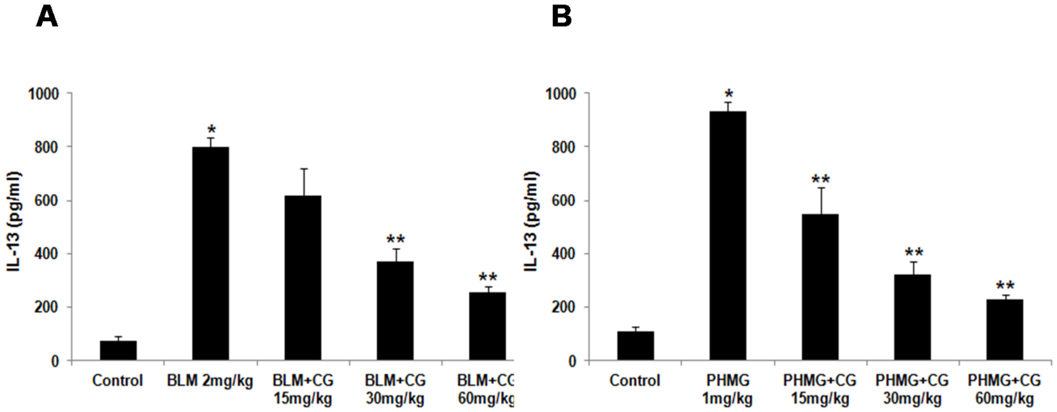

Supplementary Figure S2. CG-745 ameliorates pulmonary inflammation. The levels of IL-13 in the BALF were analyzed by ELISA. Data are presented as mean ± SD, n=5. * p<0.01 vs. control group. ** p<0.05 vs. (A) bleomycin group or (B) PHMG group.
